# Society Meetings

**Published:** 1904-01

**Authors:** 


### SOCIETY MEETINGS.

The Buffalo Academy of Medicine held meetings during the months of November and December, 1903, as follows:

*Section on Obstetrics.*—Tuesday evening, November 24. Program: Shall the ovaries be removed when operating for double pyosalpinx? C. C. Frederick; discussion opened by M. A. Crockett.

*Postponed.*—The regular meeting of this section, which should, according to the program of session, have been held Tuesday evening, December 22, was postponed.

*Section on Surgery.*—Tuesday evening, December 1. Program: Secondary syphilis and its diagnosis, Alfred E. Diehl; The use of invisible light rays in medicine and surgery, Frederick H. Millener; discussion opened by Roswell Park.

*Section on Medicine.*—Tuesday evening, December 8. Program: The diagnosis of diseases of the heart muscle, John Parmenter; the discussion led by Henry R. Hopkins; In memoriam of Dr. C. C. Wyckoff, Roswell Park, D. W. Harrington, De Lancey Rochester, memorial committee.

*Section on Pathology.*—Tuesday evening, December 15. Program: Rapid sterilisation in the flame, Marshall Clinton; A case showing trichocephalus dispar, Irving P. Lyon; Diagnosis of typhoid and colon bacilli with neutral red, George Roberts; An unusual case of amyloid degeneration, N. G. Russell.

The Medical Society of the County of Erie will hold its eighty-third annual meeting Tuesday, January 12, 1904, under the presidency of Dr. Ernest Wende, of Buffalo. The program in preparation by the secretary, Dr. F. C. Gram, will soon be issued. All physicians are cordially invited.

---

The Erie County Medical Association held its quarterly meeting at the University Club, December 7, 1903, under the presidency of Dr. Allen A. Jones, of Buffalo. Dr. A. H. Briggs, of Buffalo, read a paper entitled, A consideration of different methods of conveying the infection of typhoid fever. Dr. Prescott Le Breton, also of Buffalo, presented the report of A case of Potts's Disease, limited to the atlas and axis. A memorial of Dr. William H. Jackson, of Springville, was presented through a committee consisting of Drs. Charles G. Stockton, A. A. Hubbell and A. G. Bennett. Officers were nominated to be voted at the next meeting, as follows: president, C. C. Frederick; vice-president, A. G. Bennett; secretary, D. E. Wheeler; treasurer, Adolph H. Urban.

